# 2,2′:4,4′′:4′,4′′′-Quaterpyridine: synthesis, crystal-structure description, and Hirshfeld surface analysis

**DOI:** 10.1107/S2056989023002426

**Published:** 2023-03-21

**Authors:** Stephen O. Aderinto, Jim A. Thomas, Craig C. Robertson

**Affiliations:** a Department of Chemistry, Dainton Building, University of Sheffield, Brook Hill, Sheffield S3 7HF, United Kingdom; Venezuelan Institute of Scientific Research, Venezuela

**Keywords:** crystal structure, quaterpyridine, Hirshfeld surface analysis

## Abstract

The title compound, 2,2′:4,4′′:4′,4′′′-quaterpyridine (Qtpy), C_20_H_14_N_4_, crystallizes in the triclinic *P*  space group and has half of the mol­ecule in the asymmetric unit, corresponding to 4,4′-bi­pyridine (4,4′-bpy) that serves as the building block for the mol­ecule. C_4,4′-bpy_—N—C_4,4′-bpy_ and/or N—C_4,4′-bpy_—C_4,4′-bpy_ bond-angle parameters show that the 4,4′-bpy ligands are highly rigid, displaying values lower than the linear bond angle of 180°. In the crystal, the 4,4′-bpy units are seen to be facing each other in relatively close proximity. The most important inter­actions on the Hirshfeld Surface of the compound are C—H⋯N/H⋯N—C inter­actions (constituting 10.6% and 7.6% of the total surface).

## Abstract 2

The title compound, 2,2′:4,4′′:4′,4′′′-quaterpyridine (Qtpy), C_20_H_14_N_4_, crystallizes in the triclinic *P* 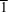 space group and has half of the mol­ecule in the asymmetric unit, corresponding to 4,4′-bi­pyridine (4,4′-bpy) that serves as the building block for the mol­ecule.

## 1. Chemical context

2,2′:4,4′′:4′,4′′′-Quaterpyridine (Qtpy) is an important bridging ligand used in synthetic inorganic chemistry for the development of many transition-metal complexes (TMCs) employed as DNA-binding probes (Morgan *et al.*, 1991 ▸; Pyle *et al.*, 1989 ▸). Previously, bridging ligands that provide low inter-metal communication (due to the absence of conjugation between two ligands subunits connected by saturated carbon chains as experienced in bridging ligands that contain isolated bi­pyridine) have been obtained by the direct fusion of two bpy moieties. However, there has been a surge in interest in ligands that can electronically and coordinatively link two metal centres. In that context, Qtpy represents one of the only instances of a ligand formed from two fused bpy units whose coordination chemistry has been widely explored (Downard *et al.*, 1991 ▸; Cooper *et al.*, 1990 ▸). 
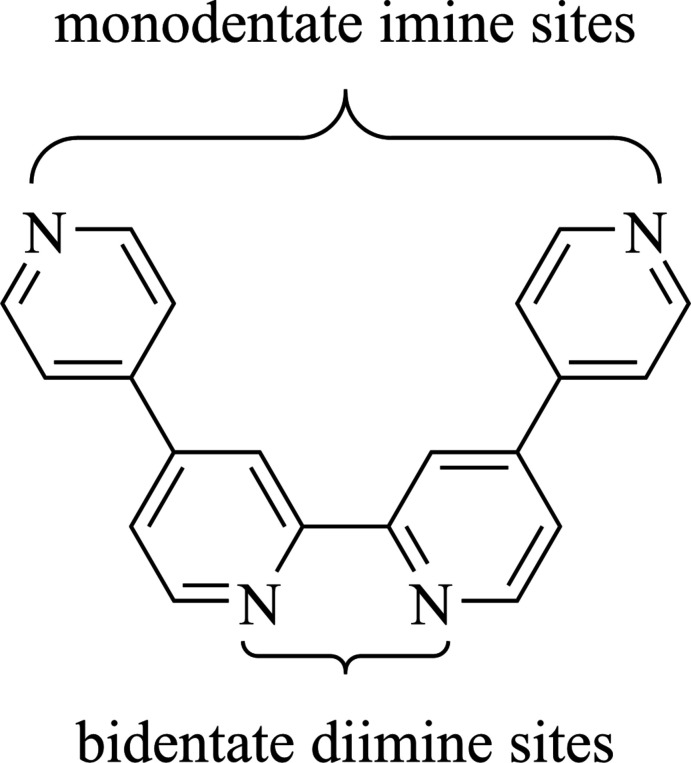


In fact, the first report of Qtpy dates back to 1938 when Burstall and colleagues obtained the ligand as a by-product of the reaction between 4,4′-bi­pyridine (4,4′-bpy) and iodine (Burstall, 1938 ▸). However, since the 1990s, studies in the use of the ligand as a building block for the construction of oligonuclear supra­molecular assemblies of photoactive and redox-active chromophoric sites have multiplied (Gorczyński *et al.*, 2016 ▸). Qtpy’s suitability for such a role arises from its possession of both a bidentate di­imine site that can coordinate through chelation to a metal centre, and also two monodentate imine sites, which can both coordinate to other metal centres (see scheme).

In a number of studies, we have employed Qtpy as a bridging ligand to synthesize novel luminescent TMCs towards therapeutic, diagnostic, theranostic and bioimaging ends. This work has mostly involved Ru^II^ and other *d* ^6^-metal ions (de Wolf *et al.*, 2006 ▸; Ghosh *et al.*, 2009 ▸; Ahmad *et al.*, 2011 ▸, 2013 ▸, 2014*a* ▸,*b* ▸; Walker *et al.*, 2016 ▸) . Despite its structural simplicity and synthetic significance, there is no report of the single-crystal structure of pure crystalline Qtpy.

## 2. Structural commentary and supra­molecular Features

**Figure 1:**
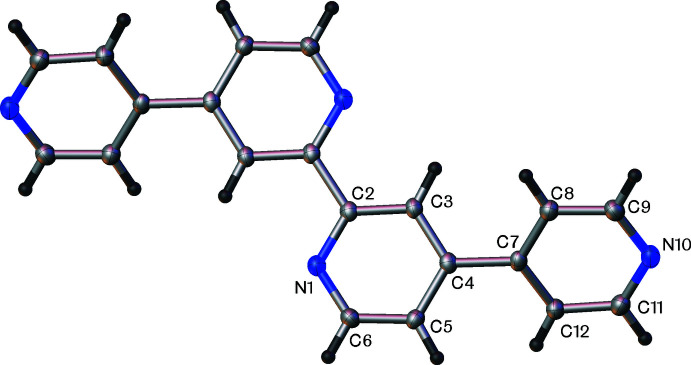
The mol­ecular structure of Qtpy showing 50% displacement ellipsoids. Half of the molecule is generated by symmetry (symmetry operation: −*x*, −*y* + 1, −*z* + 1).

**Figure 2:**
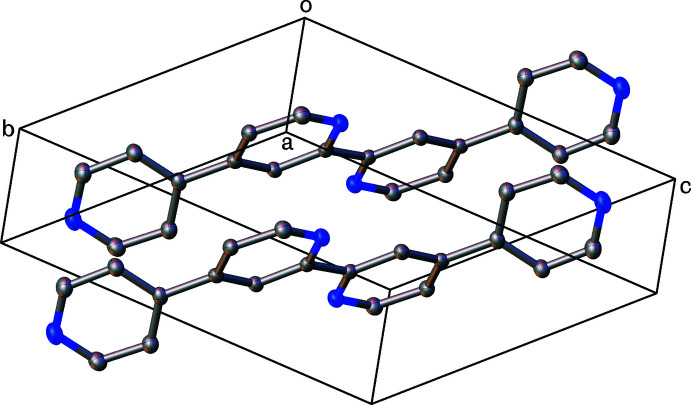
Unit cell of Qtpy with completed fragments showing the π–π stacking of the aromatic rings. Hydrogen atoms omitted for clarity.

**Figure 3:**
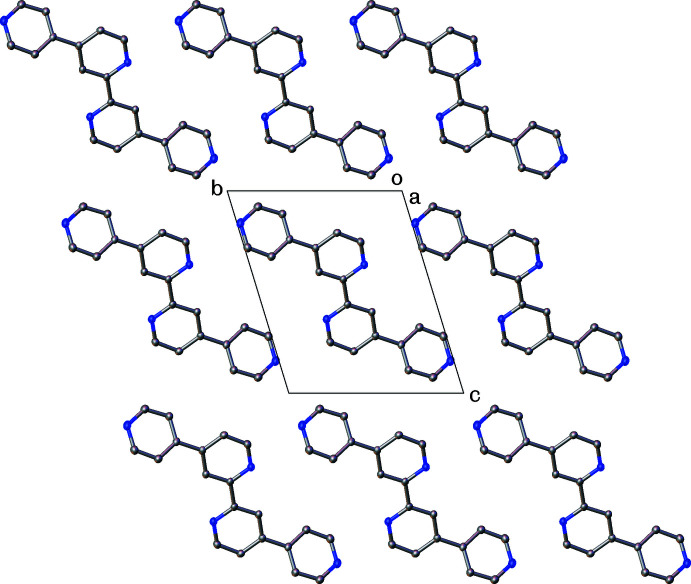
View along the *a* axis of the crystal packing showing the columnar π–π stacking through the crystal structure. Hydrogen atoms are omitted for clarity.

Qtpy (Fig. 1 ▸) crystallizes in the triclinic space group *P* 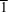 . The asymmetric unit comprises of half of a single mol­ecule, which sits on special position 1*g* (0.000, 1/2, 1/2). The 2,2′ bi­pyridine rings are planar within 0.00 (12)° and the mean torsion angle between the 4,4′-bi­pyridine rings is 34.7 (2)°. Two types of weak inter­molecular hydrogen bonds are observed between Qtpy and adjacent mol­ecules (Table 1 ▸). A single linear contact between the *sp* ^2^ hydrogen atom H9 and atom N1 of an adjacent mol­ecule (*x* + 1, *y* + 1, *z*) and a dimeric hydrogen bond between a pair of H11 and N10 atoms in a another adjacent mol­ecule (−*x* + 1, −*y* + 2, −*z*.). Both pyridine rings are engaged in π–π inter­actions (Fig. 2 ▸) between their symmetry-equivalent rings in adjacent mol­ecules, both above and below, packing in π–π-stacked columns parallel to the (100) plane (Fig. 3 ▸). The N1/C2–C6 rings pack with a distance between their centroids of 3.779 (1) Å with a shift of 1.629 Å and an angle of 0°. The C7–C9/N10/C11–C12 rings also pack with an inter­centroid distance of 3.779 (1) Å, with a shorter shift distance of 1.385 Å and an angle of 0°.

## 3. Database survey

Qtpy is a bridging ligand used in synthetic inorganic chemistry popular for the development of multinuclear TMCs. As such, a search in the Cambridge Structural Database (WebCSD, September 2022; Groom *et al.*, 2016 ▸) shows there are 19 reported structures of Qtpy utilized as a ligand: in all cases, the 2,2′-bi­pyridine has the *cis* configuration and thus acts as a bidentate chelating ligand. In seven of these structures, the monodentate 4-pyridine coordinates to a different metal centre. There are three crystal structures of modified Qtpy substrates, which are uncoordinated to metal centres. In each of these cases, as we see in our structure of Qtpy, the 2,2′-bi­pyridine is in the *trans* configuration, which is the lower energy conformation.

## 4. Hirshfeld Surface Analysis

A Hirshfeld surface analysis (HSA) was undertaken and fingerprint plots for Qtpy were generated using *Crystal Explorer 21.5* (Spackman *et al.*, 2021 ▸). HSA is an established technique to understand the various inter­molecular inter­actions present in a compound and qu­antify weak inter­actions. In mapping such inter­actions, inter­nal consistency is highly crucial when comparing structures. As such, all reported Hirshfeld surfaces reported herein have their bond lengths set to hydrogen atoms are set to typical neutron values (C—H = 1.083 Å, N—H = 1.009 Å and O—H = 0.98 3Å). A Hirshfeld surface is unique for a given crystal structure and a set of spherical atomic electron densities. It can help structural chemists gain additional insight into the inter­molecular inter­actions present in mol­ecular crystals (Spackman & McKinnon, 2002 ▸; Spackman & Jayatilaka, 2009 ▸). The *d*_norm_ values are mapped onto the Hirshfeld surface by using a red–blue–white colour scheme, where red signifies shorter contacts, white represents contacts around the van der Waals separation and blue indicates longer contacts (Montazerozohori *et al.*, 2016 ▸). The 2D fingerprint plot presents the decomposition of Hirshfeld surfaces into the contribution of different inter­molecular inter­actions present in a crystal structure; 2D fingerprint plots of Hirshfeld surfaces are usually given as plots of *d*_i_ against *d*_e_ (Montazerozohori *et al.*, 2016 ▸).

**Figure 4:**
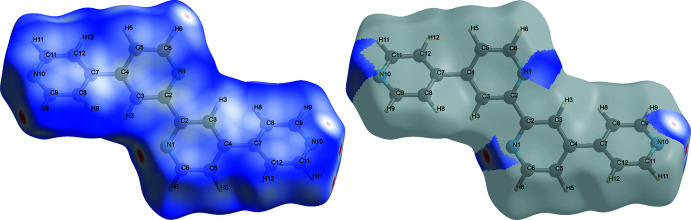
Hirshfeld surfaces of Qtpy ligand mapped over *d*_norm_ for all the inter­actions (left) and N⋯H/H⋯N inter­actions (right).

**Figure 7:**
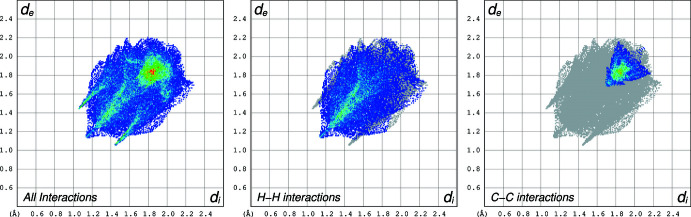
Two-dimensional fingerprint plots for the Qtpy ligand for all the inter­actions (left), H⋯H inter­actions (middle) and C⋯C inter­actions (right).

Hirshfeld surfaces of Qtpy ligand are given in Figs. 4 ▸–6 ▸ ▸ and two-dimensional fingerprint plots in Figs. 7 ▸ and 8 ▸. To visualize the calculated mol­ecular structure, the surfaces were set to be transparent (Jayendran *et al.*, 2019 ▸). The inter­molecular inter­actions (Table 2 ▸) are summarized effectively in the spots with large circular depressions (deep red) visible on the *d*_norm_ surfaces indicative of hydrogen-bonding contacts and other weak contacts. The major contact points of the inter­molecular inter­actions in the ligand involve H⋯H, as shown by the clearly visible light red spots on the *d*_norm_ surface (Hu *et al.*, 2019 ▸; Pan *et al.*, 2020 ▸). The shape-index is used to identify complementary hollows (red) and bumps (blue) where two mol­ecular surfaces touch one another. On the Hirshfeld surface mapped with the shape-index function, C—H⋯π inter­actions appear as hollow orange areas (π⋯H) and bulging blue areas (H⋯π). On the Hirshfeld surface mapped with shape-index for the ligand, these inter­actions manifest as hollow orange areas and bulging blue areas. Curvedness is a function of the root-mean-square curvature of the surface, and maps of curvedness typically show large regions of green (relatively flat) separated by dark blue edges (large positive curvature). The π–π stacking inter­actions are further evidenced by the appearance of flat surfaces towards the bottom of the compound as clearly visible on the curvedness surface.

## 5. Synthesis and crystallization

**Figure 9:**
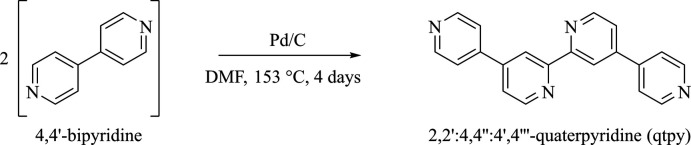
Reaction scheme to synthesize Qtpy.

Qtpy was synthesized (Fig. 9 ▸) according to the published method given by Morgan & Baker (1990 ▸). 4,4′-bpy (20.42 g, 70.19 mmol) was weighed into a 500 mL two-neck round-bottom flask to which fresh Pd/C (2.20 g) was added. DMF (300 mL) that had been deaerated for *ca* 15 min was then transferred into the flask. The reaction was left to progress under an N_2_ atmosphere while being refluxed at 426 K for *ca* 120 h. Once the reaction was complete and the mixture had cooled down to room temperature, DMF was removed by rotary evaporation to afford a mass of black residue. Chloro­form (100 mL) was added to the black residue, and the mixture was allowed to reflux under stirring for a further *ca* 30 min. Once cooled, the Pd/C catalyst was filtered off through celite to yield a clear yellow solution. Afterwards, chloro­form was removed *in vacuo* and the crude mass obtained was left to stir in acetone (60 mL) for *ca* 30 min to remove any unreacted 4,4′-bpy. The mixture was filtered under vacuum, and the residue was collected. The filtrate was concentrated by rotary evaporation to yield more portions of the desired product. There were several repetitions of this process, and the various portions of the product were reunited. The compound obtained was then recrystallized from EtOH to yield crystals of Qtpy ligand 6.84 g (33.7%) as a creamy solid but sometimes an off-white solid. ^1^H NMR (400 MHz, d_3_-CDCl_3_): δ_H_ = 8.85 (*dd*, *J* = 5.1, 2H), 8.81– 8.79 (*m*, 6H), 7.71 (*dd*, *J* = 4.5, 1.6 Hz, 4H), 7.63 (*dd*, *J* = 5.1, 1.8 Hz, 2H). ESI–MS, *m*/*z*: 311 [*M*H]^+^.

## 6. Refinement

Crystal data, data collection and structure refinement details are summarized in Table 3 ▸. Hydrogen atoms were placed in calculated positions with idealized geometries C—H = 0.95 Å) and then refined using a riding model and isotropic displacement parameters [*U*_iso_(H) = 1.2*U*_eq_(C)].

## Supplementary Material

Crystal structure: contains datablock(s) I. DOI: 10.1107/S2056989023002426/zn2024sup1.cif

Structure factors: contains datablock(s) I. DOI: 10.1107/S2056989023002426/zn2024Isup2.hkl

CCDC reference: 2248236

Additional supporting information:  crystallographic information; 3D view; checkCIF report

## Figures and Tables

**Figure 5:**
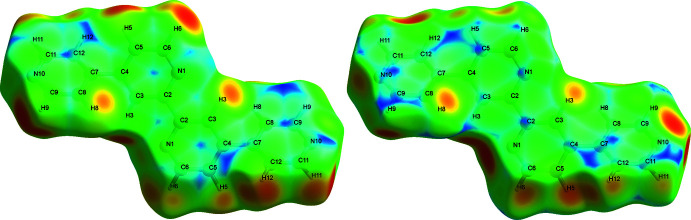
Hirshfeld surfaces of Qtpy ligand mapped with *d*_i_ (left) and *d*_e_ (right) for all the inter­actions.

**Figure 6:**
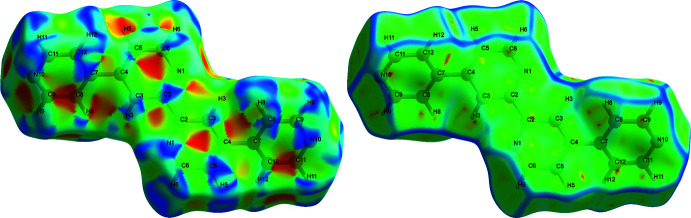
Hirshfeld surfaces of Qtpy ligand mapped with shape index (left) and curvedness (right) for all the inter­actions.

**Figure 8:**
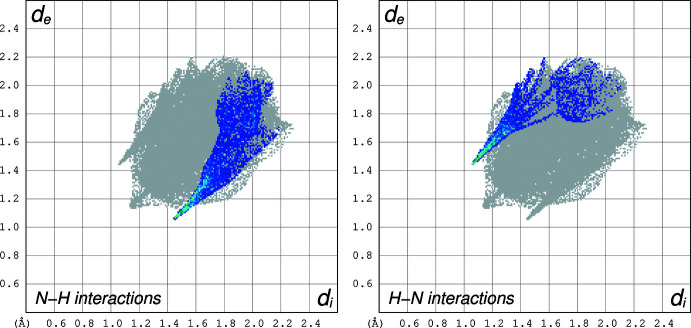
Two-dimensional fingerprint plots for the Qtpy ligand for N⋯H inter­action (left) and H⋯N inter­actions (right).

**Table 1:** Hydrogen-bond geometry (Å, °)

*D*—H⋯*A*	*D*—H	H⋯*A*	*D*⋯*A*	*D*—H⋯*A*
C9—H9⋯N1^i^	0.95	2.60	3.420 (2)	144
C11—H11⋯N10^ii^	0.95	2.62	3.410 (2)	141

Symmetry codes: (i) 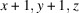 ; (ii) 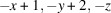 .

**Table 2:** Summary of the percentages of inter­molecular contacts contributed to the HSA surface of Qtpy ligand

Inside atom		Outside atom		Total contributions
	N	C	H	
C	3.2	15.5	6.7	25.5
H	7.6	4.2	48.5	60.4
N	0.4	3.1	10.6	14.2
Total contributions	11.2	22.8	65.8	

**Table 3:** Experimental details

Crystal data	
Chemical formula	C_20_H_14_N_4_
*M*_r_	310.35
Crystal system, space group	Triclinic, *P* 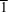
Temperature (K)	110
*a*, *b*, *c* (Å)	3.7794 (9), 9.132 (2), 11.115 (3)
α, β, γ (°)	106.477 (2), 96.768 (2), 92.720 (2)
*V* (Å^3^)	363.98 (15)
*Z*	1
Radiation type	Mo *K*α
μ (mm^−1^)	0.09
Crystal size (mm)	0.4 × 0.35 × 0.15

Data collection	
Diffractometer	Bruker APEXII CCD
Absorption correction	Multi-scan (*SADABS*; Krause *et al*., 2015 ▸)
*T*_min_, *T*_max_	0.689, 0.746
No. of measured, independent and observed [*I* > 2σ(*I*)] reflections	6826, 1617, 1176
*R*_int_	0.036
(sin θ/λ)_max_ (Å^−1^)	0.644

Refinement	
*R*[*F* ^2^ > 2σ(*F* ^2^)], *wR*(*F* ^2^), *S*	0.047, 0.125, 1.07
No. of reflections	1617
No. of parameters	109
H-atom treatment	H-atom parameters constrained
Δρ_max_, Δρ_min_ (e Å^−3^)	0.32, −0.27

Computer programs: *APEX2* (Bruker, 2016 ▸), *SAINT* (Bruker, 2016 ▸), *SHELXT* (Sheldrick, 2015*a* ▸), *SHELXL* (Sheldrick, 2015*b* ▸) and *OLEX2* (Dolomanov *et al.*, 2009 ▸).

## supplementary crystallographic information

### Crystal data

**Table:**

C_20_H_14_N_4_	*Z* = 1
*M**_r_* = 310.35	*F*(000) = 162
Triclinic, *P*1	*D*_x_ = 1.416 Mg m^−^^3^
*a* = 3.7794 (9) Å	Mo *K*α radiation, λ = 0.71073 Å
*b* = 9.132 (2) Å	Cell parameters from 1538 reflections
*c* = 11.115 (3) Å	θ = 2.3–27.1°
α = 106.477 (2)°	µ = 0.09 mm^−^^1^
β = 96.768 (2)°	*T* = 110 K
γ = 92.720 (2)°	Plate, colourless
*V* = 363.98 (15) Å^3^	0.4 × 0.35 × 0.15 mm

### Data collection

**Table:**

Bruker APEXII CCD diffractometer	1176 reflections with *I* > 2σ(*I*)
φ and ω scans	*R*_int_ = 0.036
Absorption correction: multi-scan (SADABS; Krause et al., 2016)	θ_max_ = 27.3°, θ_min_ = 1.9°
*T*_min_ = 0.689, *T*_max_ = 0.746	*h* = −4→4
6826 measured reflections	*k* = −11→11
1617 independent reflections	*l* = −14→14

### Refinement

**Table:**

Refinement on *F*^2^	Primary atom site location: dual
Least-squares matrix: full	Hydrogen site location: inferred from neighbouring sites
*R*[*F*^2^ > 2σ(*F*^2^)] = 0.047	H-atom parameters constrained
*wR*(*F*^2^) = 0.125	*w* = 1/[σ^2^(*F*_o_^2^) + (0.0544*P*)^2^ + 0.0983*P*] where *P* = (*F*_o_^2^ + 2*F*_c_^2^)/3
*S* = 1.07	(Δ/σ)_max_ < 0.001
1617 reflections	Δρ_max_ = 0.32 e Å^−^^3^
109 parameters	Δρ_min_ = −0.27 e Å^−^^3^
0 restraints	

### Special details

**Table:**

Geometry. All esds (except the esd in the dihedral angle between two l.s. planes) are estimated using the full covariance matrix. The cell esds are taken into account individually in the estimation of esds in distances, angles and torsion angles; correlations between esds in cell parameters are only used when they are defined by crystal symmetry. An approximate (isotropic) treatment of cell esds is used for estimating esds involving l.s. planes.
Refinement. A crystal with dimensions 0.1 x 0.3 x 0.3?mm was selected and intensity data was collected on a Bruker SMART APEX-II CCD diffractometer operating with a MoKα sealed-tube X-ray source of the crystal mounted in fomblin oil on a MicroMount (MiTeGen, USA) and cooled to 110?K in a stream of cold nitrogen gas using an Oxford Cryosystems 700 Cryostream. Data were corrected for absorption using empirical methods (SADABS; Bruker, 2016) based upon symmetry equivalent reflections combined with measurements at different azimuthal angles (Krause *et al.*, 2015). The crystal structures were solved and refined against F2 values using ShelXT (Sheldrick, 2015a) for solution and ShelXL (Sheldrick, 2015b) for refinement accessed via the Olex2 program (Dolomanov *et al.*, 2009). Non-hydrogen atoms were refined anisotropically. The Qtpy structure displayed here has been refined anisotropically with Final R indexes [I>2σ (I)] value of 0.0473.

### Fractional atomic coordinates and isotropic or equivalent isotropic displacement parameters (Å^2^)

**Table:**

	*x*	*y*	*z*	*U*_iso_*/*U*_eq_	
N1	0.0725 (4)	0.34513 (15)	0.36534 (13)	0.0199 (3)	
N10	0.5896 (4)	0.98051 (16)	0.15939 (14)	0.0255 (4)	
C2	0.0647 (4)	0.48982 (18)	0.43797 (14)	0.0172 (4)	
C3	0.1688 (4)	0.61573 (18)	0.40043 (15)	0.0181 (4)	
H3	0.163519	0.716337	0.455210	0.022*	
C4	0.2805 (4)	0.59466 (18)	0.28298 (15)	0.0177 (4)	
C5	0.2848 (4)	0.44507 (18)	0.20739 (15)	0.0190 (4)	
H5	0.357380	0.425003	0.125703	0.023*	
C6	0.1820 (4)	0.32596 (18)	0.25263 (15)	0.0206 (4)	
H6	0.189793	0.224202	0.200326	0.025*	
C7	0.3904 (4)	0.72786 (18)	0.24004 (15)	0.0181 (4)	
C8	0.5635 (4)	0.86115 (18)	0.32469 (16)	0.0213 (4)	
H8	0.616125	0.868956	0.412099	0.026*	
C9	0.6585 (5)	0.98241 (19)	0.28053 (16)	0.0238 (4)	
H9	0.779673	1.072268	0.339663	0.029*	
C11	0.4220 (5)	0.85210 (19)	0.07885 (17)	0.0240 (4)	
H11	0.370005	0.847837	−0.007860	0.029*	
C12	0.3199 (4)	0.72498 (19)	0.11441 (16)	0.0213 (4)	
H12	0.201979	0.636146	0.052993	0.026*	

### Atomic displacement parameters (Å^2^)

**Table:**

	*U*^11^	*U*^22^	*U*^33^	*U*^12^	*U*^13^	*U*^23^
N1	0.0216 (8)	0.0177 (7)	0.0212 (8)	0.0007 (6)	0.0036 (6)	0.0068 (6)
N10	0.0314 (9)	0.0205 (8)	0.0279 (9)	0.0025 (6)	0.0094 (7)	0.0102 (7)
C2	0.0159 (8)	0.0174 (8)	0.0187 (9)	0.0013 (6)	−0.0005 (6)	0.0073 (7)
C3	0.0179 (9)	0.0161 (8)	0.0205 (9)	−0.0001 (6)	0.0010 (7)	0.0068 (7)
C4	0.0150 (8)	0.0174 (8)	0.0212 (9)	−0.0003 (6)	0.0003 (6)	0.0073 (7)
C5	0.0190 (9)	0.0210 (9)	0.0178 (9)	0.0001 (7)	0.0028 (7)	0.0071 (7)
C6	0.0235 (9)	0.0163 (8)	0.0215 (9)	0.0016 (7)	0.0037 (7)	0.0042 (7)
C7	0.0175 (9)	0.0174 (8)	0.0218 (9)	0.0032 (6)	0.0058 (7)	0.0082 (7)
C8	0.0234 (9)	0.0207 (9)	0.0210 (9)	0.0018 (7)	0.0041 (7)	0.0076 (7)
C9	0.0266 (10)	0.0188 (9)	0.0253 (9)	−0.0003 (7)	0.0049 (7)	0.0051 (7)
C11	0.0293 (10)	0.0227 (9)	0.0220 (9)	0.0031 (7)	0.0065 (7)	0.0085 (7)
C12	0.0246 (9)	0.0186 (9)	0.0212 (9)	−0.0003 (7)	0.0041 (7)	0.0067 (7)

### Geometric parameters (Å, º)

**Table:**

N1—C2	1.343 (2)	C5—C6	1.379 (2)
N1—C6	1.333 (2)	C6—H6	0.9500
N10—C9	1.336 (2)	C7—C8	1.387 (2)
N10—C11	1.333 (2)	C7—C12	1.383 (2)
C2—C2^i^	1.482 (3)	C8—H8	0.9500
C2—C3	1.385 (2)	C8—C9	1.381 (2)
C3—H3	0.9500	C9—H9	0.9500
C3—C4	1.384 (2)	C11—H11	0.9500
C4—C5	1.388 (2)	C11—C12	1.381 (2)
C4—C7	1.486 (2)	C12—H12	0.9500
C5—H5	0.9500		
			
C6—N1—C2	117.10 (13)	C5—C6—H6	118.0
C11—N10—C9	116.52 (14)	C8—C7—C4	121.35 (15)
N1—C2—C2^i^	116.76 (17)	C12—C7—C4	121.41 (15)
N1—C2—C3	122.60 (15)	C12—C7—C8	117.23 (14)
C3—C2—C2^i^	120.64 (18)	C7—C8—H8	120.4
C2—C3—H3	120.1	C9—C8—C7	119.30 (15)
C4—C3—C2	119.86 (15)	C9—C8—H8	120.4
C4—C3—H3	120.1	N10—C9—C8	123.72 (16)
C3—C4—C5	117.50 (14)	N10—C9—H9	118.1
C3—C4—C7	120.90 (15)	C8—C9—H9	118.1
C5—C4—C7	121.60 (14)	N10—C11—H11	118.2
C4—C5—H5	120.5	N10—C11—C12	123.68 (16)
C6—C5—C4	119.02 (15)	C12—C11—H11	118.2
C6—C5—H5	120.5	C7—C12—H12	120.2
N1—C6—C5	123.91 (15)	C11—C12—C7	119.54 (16)
N1—C6—H6	118.0	C11—C12—H12	120.2

Symmetry code: (i) −*x*, −*y*+1, −*z*+1.

### Hydrogen-bond geometry (Å, º)

**Table:**

*D*—H···*A*	*D*—H	H···*A*	*D*···*A*	*D*—H···*A*
C9—H9···N1^ii^	0.95	2.60	3.420 (2)	144
C11—H11···N10^iii^	0.95	2.62	3.410 (2)	141

Symmetry codes: (ii) *x*+1, *y*+1, *z*; (iii) −*x*+1, −*y*+2, −*z*.

### 1

**Table:**

Inside Atom	Outside Atom	Total Contributions			
	N	C	H		
C	3.2	15.5	6.7	25.4	
H	7.6	4.2	48.5	60.3	
N	0.4	3.1	10.6	14.1	
Total Contributions	11.2	22.8	65.8		
